# Supplementary material for: The Effect of a Structured Intervention to Improve Motor Skills in Preschool Children: Results of a Randomized Controlled Trial Nested in a Cohort Study of Danish Preschool Children, the MiPS Study
Source: Int J Environ Res Public Health. 2021 Nov 23;18(23):12272. doi: 10.3390/ijerph182312272 (PMC8656792; doi:10.3390/ijerph182312272)
Supplement: Supplementary file 1 [file ijerph-18-12272-s001.zip › File S1.pdf]

File S1 Description of the project by the Municipality of Svendborg

Definition of Gazelle Children's House and Gazelle Daycare
The participating children's houses and daycare centres are referred to as Gazelle Children's Houses and Gazelle Daycare Centres respectively, and are defined as places where there is a particular focus on physical activity and the use of physical activity as a pedagogical tool. Physical activity is thus used both as an activity with a targeted focus on children's physical development and as a fun and motivating tool to achieve other learning objectives.

It is important to note that the age span of children in daycare is large. Therefore, the motor challenges and intensity of activities must be adapted to the individual child's level of development. We call it *development-oriented educational sport*.

In addition to this general description are the following specific requirements for the participating Gazelle Children's Houses and Gazelle Daycare Centres.

The five Gazelle leaps

'Gazelle leaps' is the name given to the elements that the Gazelle Children's Houses and Gazelle Daycare Centres must meet:

1. At least four days a week, and preferably five, a minimum of 45-minute adult-initiated and adult-led activities must be carried out, where all children participate. During the week, all bodily skills mentioned below must be challenged.
2. The majority of the planned activities must be carried out outdoors.
3. The body and movement must be used as an educational tool in working with other learning-level themes - called educational sport.
4. All children must have an elevated pulse every day
5. The individual daycare centre must develop learning environments in accordance with the description *Learning Environments in Active Children in Daycare - healthier, happier and smarter children*

1. At least four days a week, and preferably five, a minimum of 45-minute adult-initiated and adult-led activities must be carried out, where all children participate. During the week, the following bodily skills must be challenged:

Bodily skills	What and how ? (examples)	Why? (examples)
Motor function	*Gross motor challenges* such as creeping, crawling, running, jumping, hopscotch, jumping, climbing<br><br>*Fine motor challenges* such as holding a pencil, handling small objects like beads and construction toys or catching insects<br><br>*Coordination Exercises* such as crawling exercises, cross-body movements, "Angels in the Snow", jumping jack and throwing, gripping and kicking exercises. Other examples can be rhythm and dance.<br><br>Different dynamic and static *balance exercises* such as walking on a line and standing on one leg.	Gross and fine motor skills are important because they form the basis of many everyday activities and are an important factor for the child's enjoyment of physical activity and thereby also the amount of the child's physical expression.<br><br>Coordination is the foundation for a long series of specific skills such as throwing and catching and many everyday activities such as pouring water into a glass.<br><br>A good balance is, amongst other things, important in relation to avoiding falls and injuries, and affects many daily activities such as putting on clothes and shoes.
Sensing	Challenges of the following senses:<br><br>The *vestibular* sense is stimulated for example by rolling, turning around, doing somersaults and swinging.<br><br>The *tactile* sense is stimulated by touch from others, for example, in the form of massage and by touching various materials and objects of different size, shape and temperature.<br><br>The *kinaesthetic* sense is stimulated by challenging the body's joints, muscles and tendons in different ways, for example, by bending, stretching and pushing, lifting objects of different weights and by fast and slow movements.	We use all of our senses to collect and process the information and experiences that we give our body and our brain. The senses are thus to control and develop our balance, coordination and motor skills and are thereby important to be able to perform both defined work routines and everyday activities.
Relaxation	The children will also experience other types of physical stimulus, namely relaxation and unwinding. It can, for example, be through massage, children's yoga or similar.	Relaxation is a good counterpart to dynamic activity, which together promote body consciousness in children. At the same time, relaxation helps to create calmer children and fewer conflicts.

It is important to point out that there is a close link, and also a direct overlap, between the different bodily skills. Therefore, an activity may contain elements of several skills. For example, exercises that stimulate the vestibular sense often also contain elements of balance.

The individual children's houses and daycare centres organize the specific activities themselves. In mixed groups of children of various ages, it is recommended that, where possible, to divide the children up, so that the groups are about the same developmental level. It provides greater opportunity to target the activity to the individual child and less need for differentiation of activities.

2. The majority of the planned activities must be carried out outdoors
Life outdoors generally creates more physical activity and better body awareness among children. This means the children's physique is challenged more because nature encourages and even requires that children crawl, jump, roll or balance.

3. Educational sport: body and movement is used as an educational tool in working with other curriculum themes

Educational sport is the name of an educational approach which uses physical activity as a tool to achieve educational goals that are not necessarily related to children's physical and motor development. Physical activity, play, movement and motor development are inextricably linked when you talk about children in daycare. Both the children's desire to move and the level of physical activity depend very much on how good their motor skills are. In addition, motor skills have a number of other major positive effects, amongst other things, on the child's self-esteem and social competence. Physical activity is thus a teaching tool that can be used to promote physical, mental, social and cognitive development in children. This is due primarily to bodily experiences that are better retained in the mind and the body's subconscious, because they are experienced by several of the body's senses at the same time. When the body is in use, several centers of the brain are awakened, which increases learning. Children who have a good understanding of the body will also be more familiar with their body and have better motor skills and more highly stimulated senses, which means that they have more capacity to focus on the surroundings, the social relationships or the cognitive challenges that they face when they play and learn.

4. All children must have an elevated pulse every day
A normal day in daycare contains a variety of activities that strengthen various bodily skills. At the same time, we know that there is a correlation between the intensity of physical activity one develops and its health effects, and that physical activity of high intensity has the greatest effects. Therefore, all children in the Gazelle Children’s Houses and Gazelle Childcare Centres must have an elevated pulse every day.

5. The individual daycare centre must develop learning environments in accordance with the *Learning Environments in Active Children in Daycare*, which is published as an appendix to this concept.

The decor of the learning environments through which children move greatly affects their behaviour. Therefore, it is important that learning environments are created where the employees and the decor are conducive to physical activity, and where physical activity is recognized as something positive and used as an educational tool.

The employees' role and training
Children's natural behaviour is largely determined by whatever gives them pleasure. Therefore, as a rule, children play the games they want. This means that the daycare centre employees have a task in creating motivation amongst children so that they have a natural desire to be physically active. It is all about introducing games and activities that create joy and give children a sense of mastery. There must be a culture where there is room to experiment and practise. This applies to both children and adults. Employees must challenge their own physicality and lead the way as role models who will, can and dare.

At the same time, centre employees have the task of involving the body and movement as an educational tool and of developing activities that address and involve all children.

All employees have to undergo a 37-hour competency development program with a view to making this concept work in reality. The competency development program will be described in a separate document which will, amongst other things, contain knowledge about children's physical and sensory-motor development, activity development, motivation and the use of physical activity as a pedagogical tool.

Parental Involvement
The parents are kept informed about the project on a current basis, and a separate description of the project is prepared that targets the parents. Each Gazelle Children's House and the individual Gazelle Childcare Centre handles the concrete and practical information on the project, and creates a continuous and constructive dialogue with the parents about the child's well-being, development, learning and upbringing.

Organization
*The project is an interdisciplinary project between the discipline areas of Children and Youth, Social and Health, Culture, Business and Development. Children and Youth handle the coordination of the project.
*The project is politically anchored in the Committee for Children and Youth, the Social and Health Committee and the Business, Employment and Culture Committee.
*Each Gazelle Children's House and Childcare Centre appoints coordinators who participate in a network and function as contact people.
*The teaching team leaders in the Gazelle Children's Houses and Childcare Centres participate in a specially organized competency development program
*The teaching team leaders in the Gazelle Children's Houses and Childcare Centres must implement a process in the employee group that relates to preparing a script, which as a minimum:

- describes how the concept is implemented in each Gazelle Children's House and individual Gazelle Childcare Centre

- describes how the five Gazelle leaps are implemented. A working example of a weekly schedule is prepared
- describes how parents are involved in and informed about the project.

Sharing of knowledge

The designated coordinators can be both managers and employees. Coordinators participate in regular networking meetings where ideas are shared and the latest information on the project is provided. During the project start-up, the network meets every second month. Besides the network, an idea bank is created where employees can find and share inspiration for activity development and creative ideas. There must be opportunities to share pictures and videos.

Gazelle Children's Houses are also encouraged to occasionally utilize each other's employees in order to exploit special skills and interests, and in this way, create an even more varied offering of activities for the children.

In addition, it is recommended that daycare centres make joint investments in portable tools that can rotate between the different centres and children's houses.
