# Supplementary material for: The Effect of a Structured Intervention to Improve Motor Skills in Preschool Children: Results of a Randomized Controlled Trial Nested in a Cohort Study of Danish Preschool Children, the MiPS Study
Source: Int J Environ Res Public Health. 2021 Nov 23;18(23):12272. doi: 10.3390/ijerph182312272 (PMC8656792; doi:10.3390/ijerph182312272)
Supplement: Supplementary file 1 [file ijerph-18-12272-s001.zip › File S2.pdf]

File S2. Scatter plots of change scores at follow-up vs. baseline values for each child, stratified by preschool. Within each pre-school, a regression line is fitted. Preschools from the intervention group are shown in red, preschools from the control group are shown in blue. In the title of each subgraph, the mean baseline value and the mean change score are shown for each preschool. The preschools are sorted by the mean value at baseline.

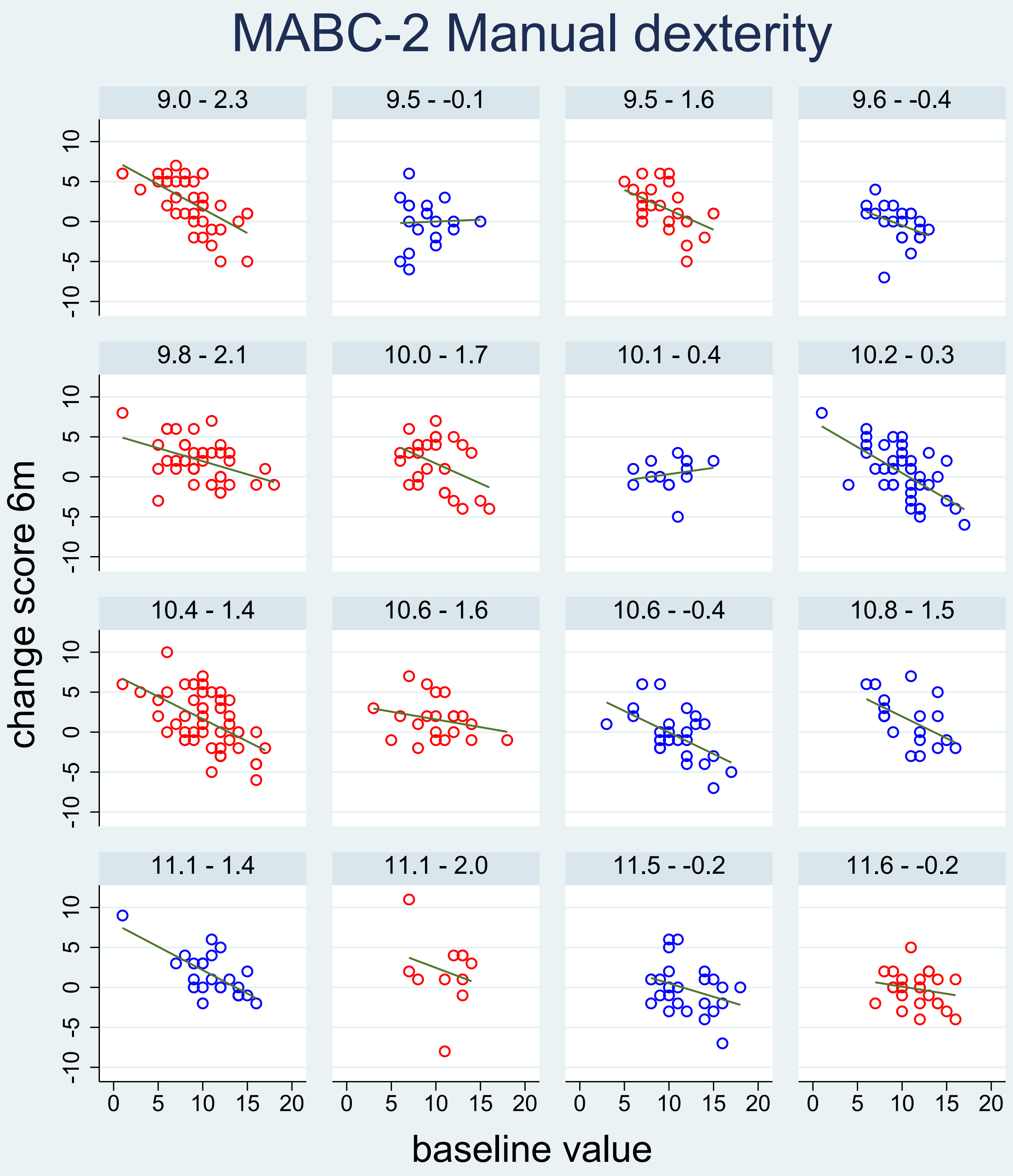
MABC-2 Manual dexterity
9.0 - 2.3
9.5 - -0.1
9.5 - 1.6
9.6 - -0.4
9.8 - 2.1
10.0 - 1.7
10.1 - 0.4
10.2 - 0.3
10.4 - 1.4
10.6 - 1.6
10.6 - -0.4
10.8 - 1.5
11.1 - 1.4
11.1 - 2.0
11.5 - -0.2
11.6 - -0.2
change score 6m
baseline value

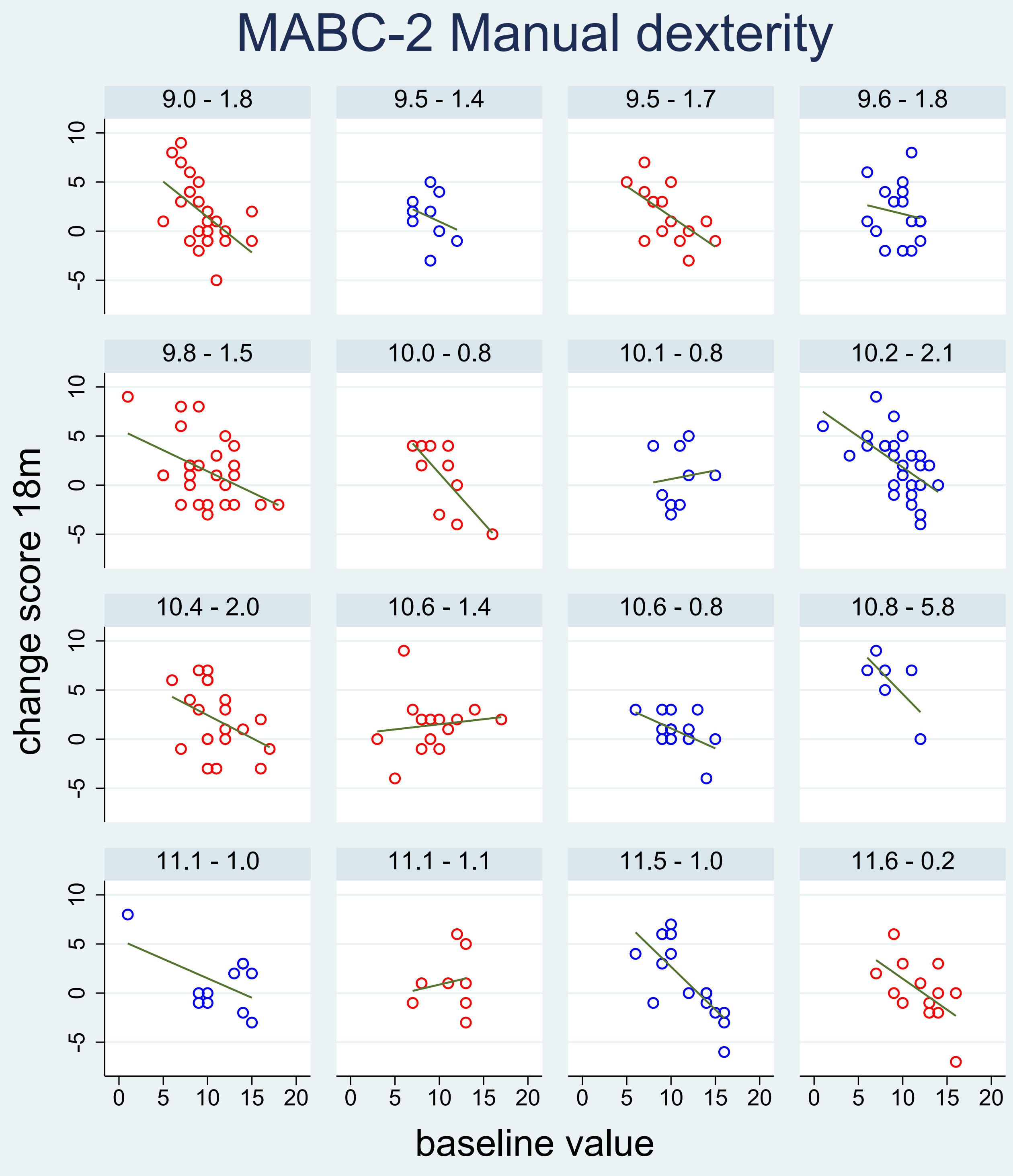

MABC-2 Manual dexterity
9.0 - 1.8
9.5 - 1.4
9.5 - 1.7
9.6 - 1.8
9.8 - 1.5
10.0 - 0.8
10.1 - 0.8
10.2 - 2.1
10.4 - 2.0
10.6 - 1.4
10.6 - 0.8
10.8 - 5.8
11.1 - 1.0
11.1 - 1.1
11.5 - 1.0
11.6 - 0.2
change score 18m
baseline value

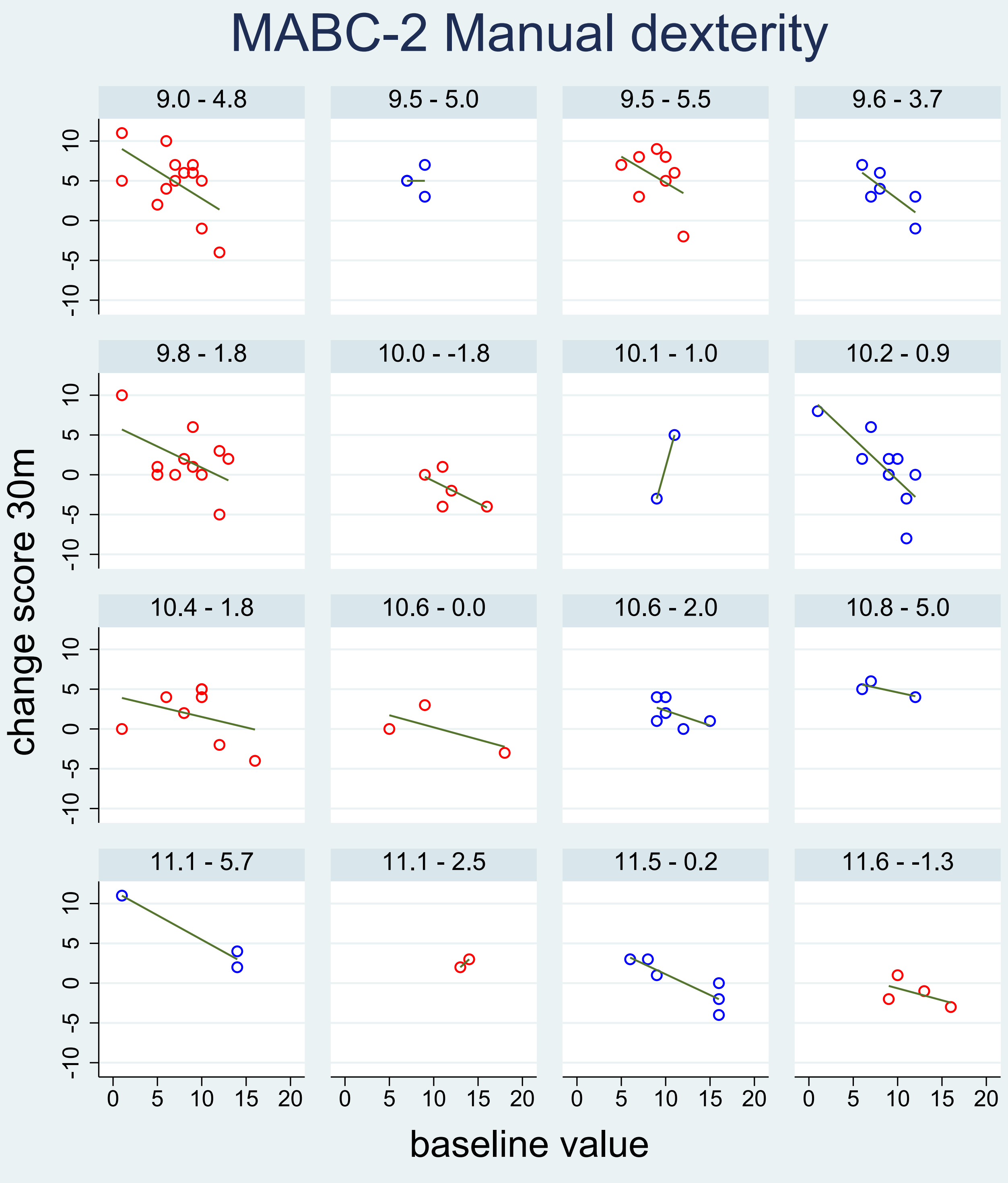
MABC-2 Manual dexterity
9.0 - 4.8
9.5 - 5.0
9.5 - 5.5
9.6 - 3.7
9.8 - 1.8
10.0 - -1.8
10.1 - 1.0
10.2 - 0.9
10.4 - 1.8
10.6 - 0.0
10.6 - 2.0
10.8 - 5.0
11.1 - 5.7
11.1 - 2.5
11.5 - 0.2
11.6 - -1.3
change score 30m
baseline value

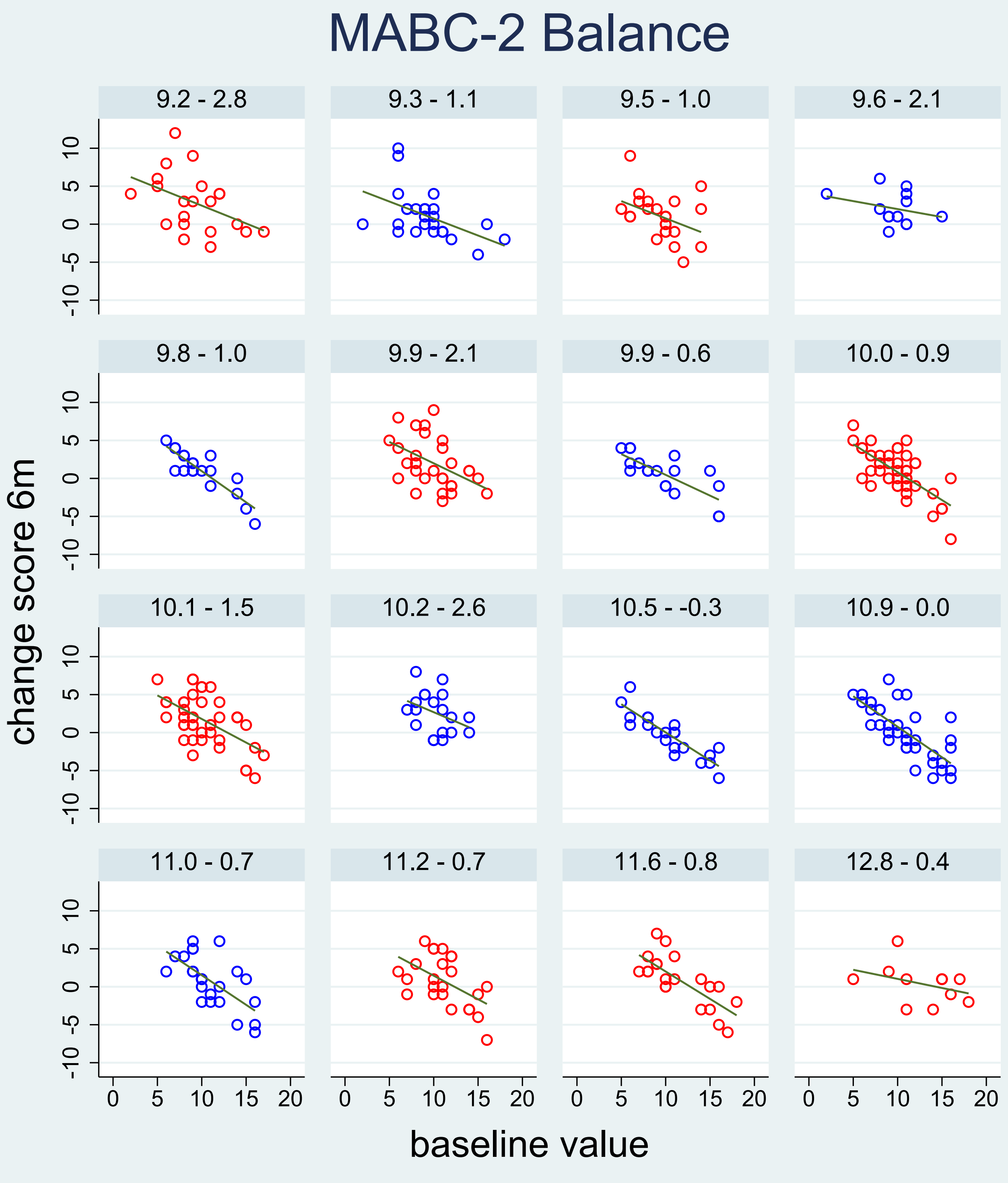

MABC-2 Balance
9.2 - 2.8
9.3 - 1.1
9.5 - 1.0
9.6 - 2.1
9.8 - 1.0
9.9 - 2.1
9.9 - 0.6
10.0 - 0.9
10.1 - 1.5
10.2 - 2.6
10.5 - -0.3
10.9 - 0.0
11.0 - 0.7
11.2 - 0.7
11.6 - 0.8
12.8 - 0.4
change score 6m
baseline value

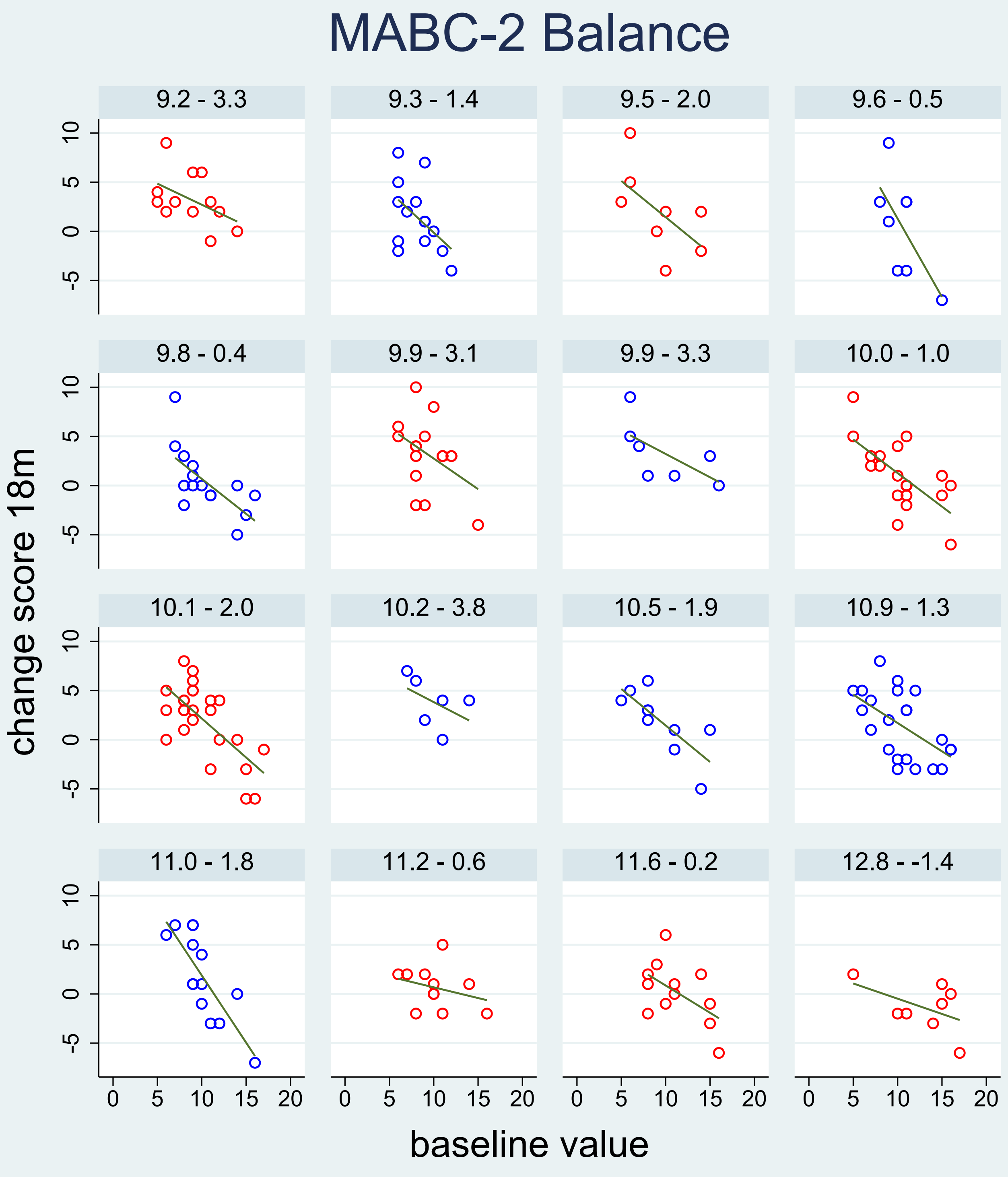
MABC-2 Balance
9.2 - 3.3
9.3 - 1.4
9.5 - 2.0
9.6 - 0.5
9.8 - 0.4
9.9 - 3.1
9.9 - 3.3
10.0 - 1.0
10.1 - 2.0
10.2 - 3.8
10.5 - 1.9
10.9 - 1.3
11.0 - 1.8
11.2 - 0.6
11.6 - 0.2
12.8 - -1.4
change score 18m
baseline value

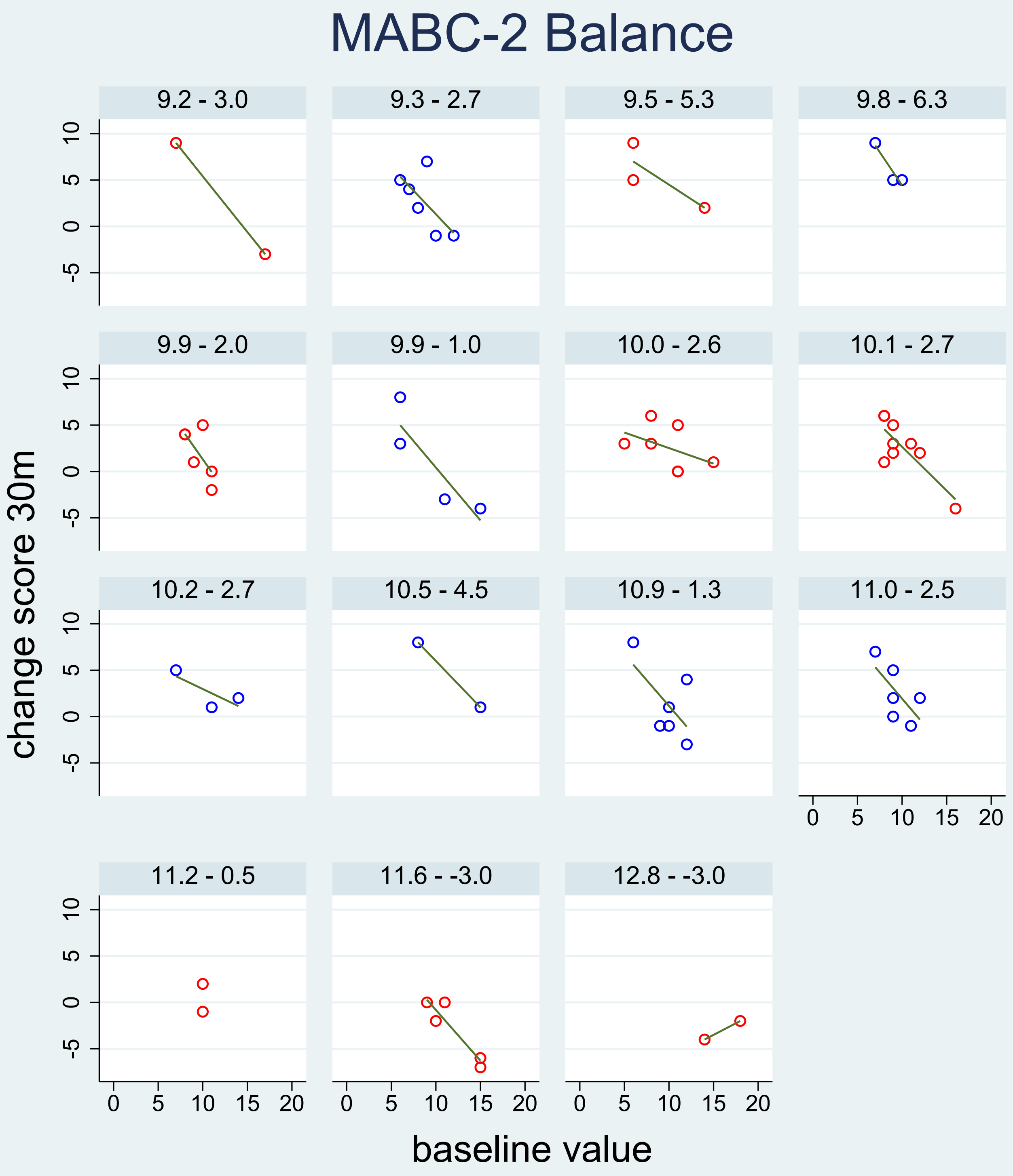

MABC-2 Balance
9.2 - 3.0
9.3 - 2.7
9.5 - 5.3
9.8 - 6.3
9.9 - 2.0
9.9 - 1.0
10.0 - 2.6
10.1 - 2.7
10.2 - 2.7
10.5 - 4.5
10.9 - 1.3
11.0 - 2.5
11.2 - 0.5
11.6 - -3.0
12.8 - -3.0
change score 30m
baseline value
-5
0
5
10
15
20

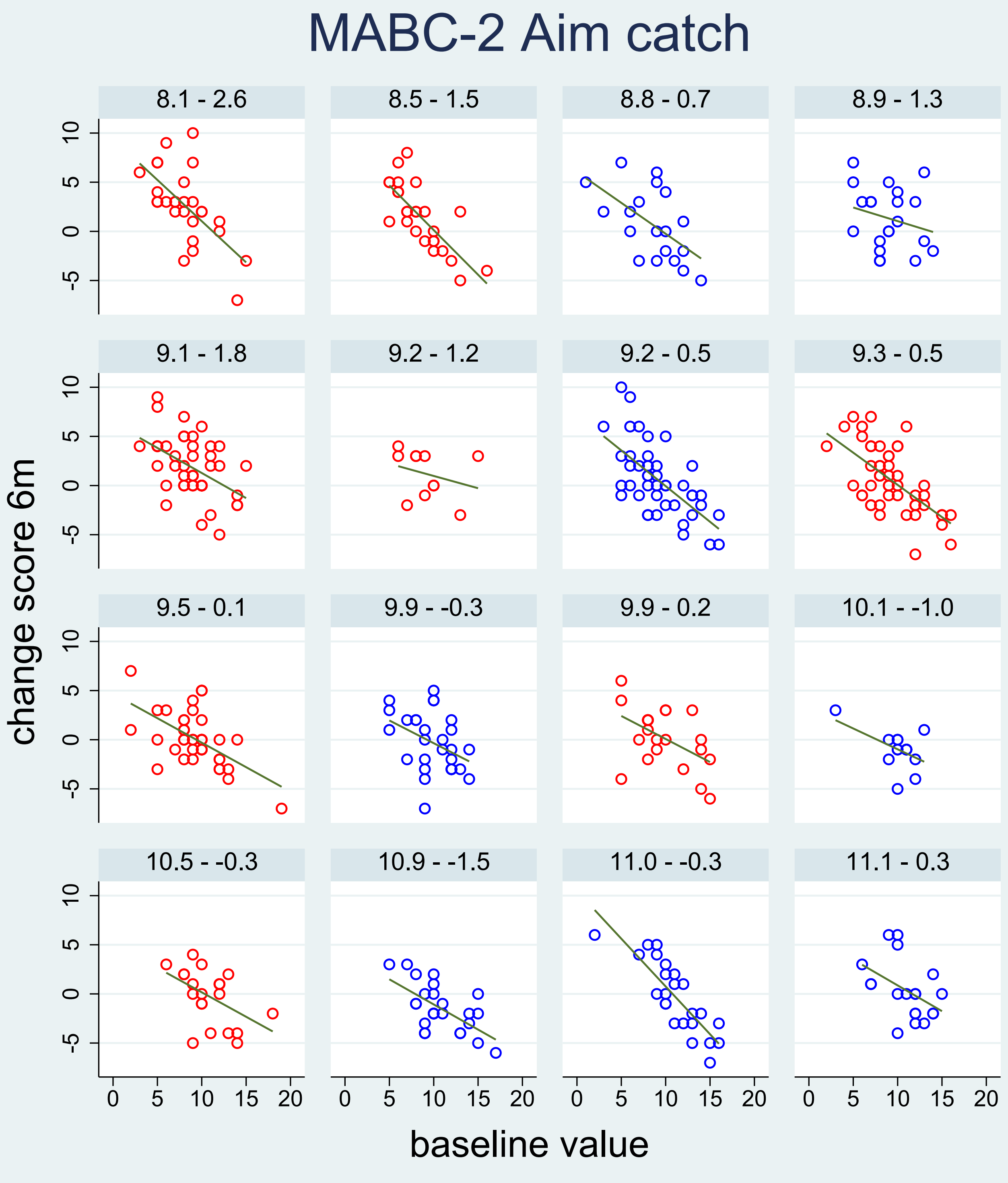

MABC-2 Aim catch
8.1 - 2.6
8.5 - 1.5
8.8 - 0.7
8.9 - 1.3
9.1 - 1.8
9.2 - 1.2
9.2 - 0.5
9.3 - 0.5
9.5 - 0.1
9.9 - -0.3
9.9 - 0.2
10.1 - -1.0
10.5 - -0.3
10.9 - -1.5
11.0 - -0.3
11.1 - 0.3
change score 6m
baseline value
-5
0
5
10
15
20

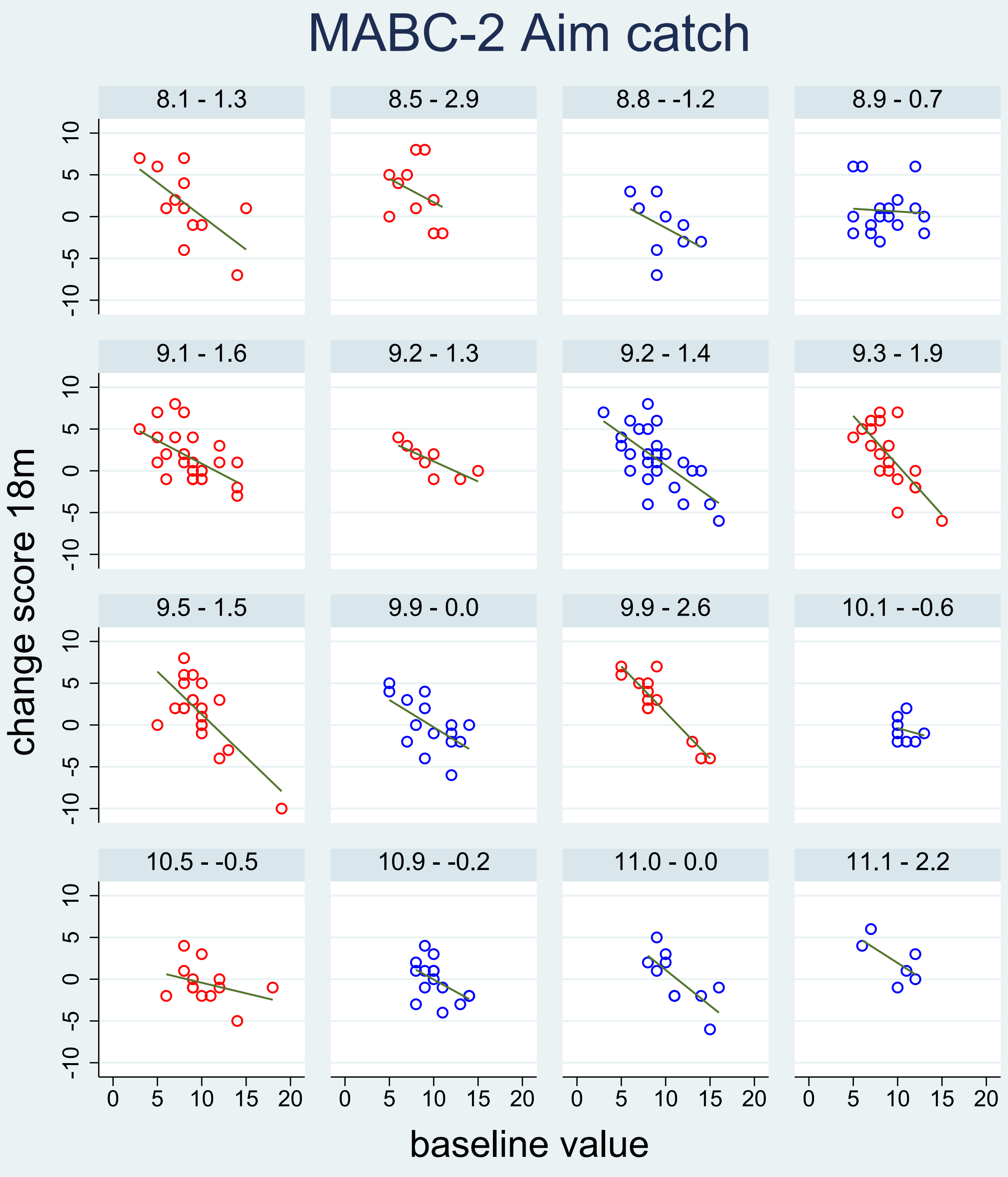
MABC-2 Aim catch
8.1 - 1.3
8.5 - 2.9
8.8 - -1.2
8.9 - 0.7
9.1 - 1.6
9.2 - 1.3
9.2 - 1.4
9.3 - 1.9
9.5 - 1.5
9.9 - 0.0
9.9 - 2.6
10.1 - -0.6
10.5 - -0.5
10.9 - -0.2
11.0 - 0.0
11.1 - 2.2
change score 18m
baseline value

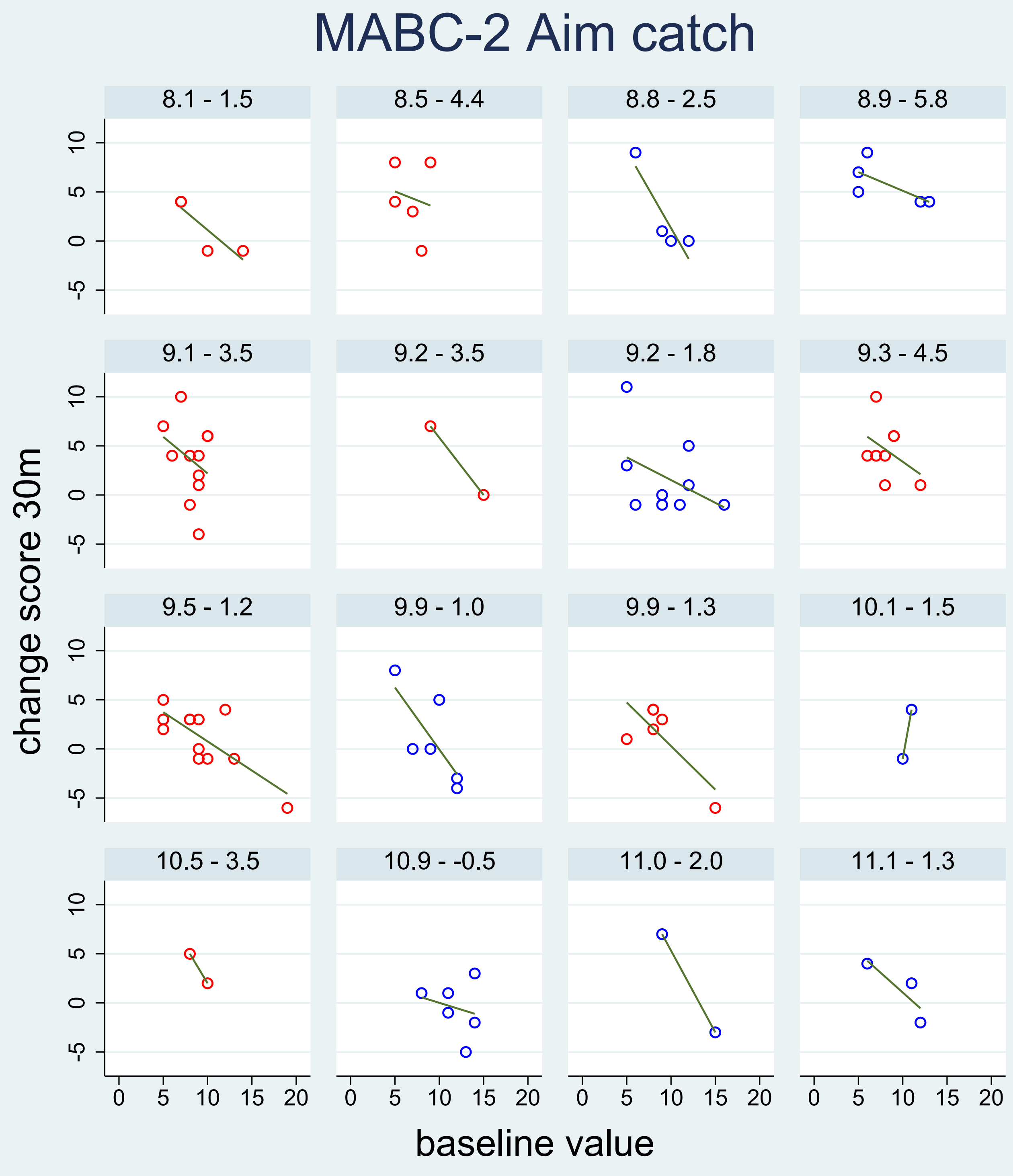
MABC-2 Aim catch
8.1 - 1.5
8.5 - 4.4
8.8 - 2.5
8.9 - 5.8
9.1 - 3.5
9.2 - 3.5
9.2 - 1.8
9.3 - 4.5
9.5 - 1.2
9.9 - 1.0
9.9 - 1.3
10.1 - 1.5
10.5 - 3.5
10.9 - -0.5
11.0 - 2.0
11.1 - 1.3
change score 30m
baseline value
